# Comparison of Natural Language Processing of Clinical Notes With a Validated Risk-Stratification Tool to Predict Severe Maternal Morbidity

**DOI:** 10.1001/jamanetworkopen.2022.34924

**Published:** 2022-10-05

**Authors:** Mark A. Clapp, Ellen Kim, Kaitlyn E. James, Roy H. Perlis, Anjali J. Kaimal, Thomas H. McCoy, Sarah Rae Easter

**Affiliations:** 1 Department of Obstetrics and Gynecology, Massachusetts General Hospital, Boston; 2 Department of Radiation Oncology, Brigham and Women’s Hospital, Boston, Massachusetts; 3 Center for Quantitative Health, Massachusetts General Hospital, Boston; 4 Department of Psychiatry, Massachusetts General Hospital, Boston; 5 Department of Population Medicine, Harvard Medical School, Boston, Massachusetts; 6 Department of Obstetrics and Gynecology, Brigham and Women’s Hospital, Boston, Massachusetts

## Abstract

**Importance:**

Risk-stratification tools are routinely used in obstetrics to assist care teams in assessing and communicating risk associated with delivery. Electronic health record data and machine learning methods may offer a novel opportunity to improve and automate risk assessment.

**Objective:**

To compare the predictive performance of natural language processing (NLP) of clinician documentation with that of a previously validated tool to identify individuals at high risk for maternal morbidity.

**Design, Setting, and Participants:**

This retrospective diagnostic study was conducted at Brigham and Women’s Hospital and Massachusetts General Hospital, Boston, Massachusetts, and included individuals admitted for delivery at the former institution from July 1, 2016, to February 29, 2020. A subset of these encounters (admissions from February to December 2018) was part of a previous prospective validation study of the Obstetric Comorbidity Index (OB-CMI), a comorbidity-weighted score to stratify risk of severe maternal morbidity (SMM).

**Exposures:**

Natural language processing of clinician documentation and OB-CMI scores.

**Main Outcomes and Measures:**

Natural language processing of clinician-authored admission notes was used to predict SMM in individuals delivering at the same institution but not included in the prospective OB-CMI study. The NLP model was then compared with the OB-CMI in the subset with a known OB-CMI score. Model discrimination between the 2 approaches was compared using the DeLong test. Sensitivity and positive predictive value for the identification of individuals at highest risk were prioritized as the characteristics of interest.

**Results:**

This study included 19 794 individuals; 4034 (20.4%) were included in the original prospective validation study of the OB-CMI (testing set), and the remaining 15 760 (79.6%) composed the training set. Mean (SD) age was 32.3 (5.2) years in the testing cohort and 32.2 (5.2) years in the training cohort. A total of 115 individuals in the testing cohort (2.9%) and 468 in the training cohort (3.0%) experienced SMM. The NLP model was built from a pruned vocabulary of 2783 unique words that occurred within the 15 760 admission notes from individuals in the training set. The area under the receiver operating characteristic curve of the NLP-based model for the prediction of SMM was 0.76 (95% CI, 0.72-0.81) and was comparable with that of the OB-CMI model (0.74; 95% CI, 0.70-0.79) in the testing set (*P* = .53). Sensitivity (NLP, 28.7%; OB-CMI, 24.4%) and positive predictive value (NLP, 19.4%; OB-CMI, 17.6%) were comparable between the NLP and OB-CMI high-risk designations for the prediction of SMM.

**Conclusions and Relevance:**

In this study, the NLP method and a validated risk-stratification tool had a similar ability to identify patients at high risk of SMM. Future prospective research is needed to validate the NLP approach in clinical practice and determine whether it could augment or replace tools requiring manual user input.

## Key Points

**Question:**

How does the predictive performance of natural language processing of clinician documentation compare with that of a previously validated tool for maternal morbidity risk stratification?

**Findings:**

In this diagnostic study of 19 794 individuals, the natural language processing–based risk-stratification method performed as well as a validated risk score for severe maternal morbidity when comparing sensitivity and positive predictive value.

**Meaning:**

The findings suggest that artificial intelligence and advanced analytics have potential applications to improve patient care while reducing non–patient-facing tasks for clinical staff.

## Teaser

This diagnostic study compares the performance of natural language processing of clinician documentation with that of a validated risk-stratification tool in assessing the risk of severe maternal morbidity.

## Introduction

Severe maternal morbidity (SMM) is a public health priority in the US, with rates exceeding those of other higher-income countries.^1,2,3,4,5^ The development and application of risk-stratification tools are important in recognizing and preventing morbidity by facilitating risk-appropriate care (ie, ensuring high-risk individuals deliver at a center with the appropriate staff and resources) and increasing health care team awareness of risk.^6^ For the prediction of SMM, prior models have focused primarily on maternal comorbidities and pregnancy characteristics, with some of the largest studies based on insurance claims data.^7,8,9,10,11,12,13,14,15,16^ These approaches, which use diagnosis codes, are limited by their inability to determine temporality between conditions and outcomes within a delivery hospitalization or understand what was known at the time of admission.^17,18^ Thus, although these diagnosis code–based risk-stratification tools tend to have good performance characteristics, their ability to be translated to clinical use is not well known. One comorbidity-based tool, the Obstetric Comorbidity Index (OB-CMI), has been successfully adapted and validated for use in clinical practice to stratify a patient’s risk of SMM during the delivery encounter.^19^

Electronic health records (EHRs), which are now ubiquitous, generate a substantial amount of digital data as a byproduct of routine clinical care. For example, most hospital admissions have a history and physical (H&P) note, usually with typed words (as opposed to a scanned image of a handwritten note) that documents a patient’s indications for admission, medical history, overall clinical assessment, and plan. Artificial intelligence tools such as natural language processing (NLP) can be applied to these data to improve identification of high-risk patients and allow for the implementation of earlier mitigation strategies, ultimately improving patient outcomes. Natural language processing is the process of computing meaning from human-authored free text, such as an H&P note, and is beginning to be used in more clinical applications.^20,21,22,23,24,25^ In this study, our objective was to compare the predictive performance of NLP of clinician documentation with that of a previously validated tool for maternal risk stratification that is currently the standard of care at the study institution.^19^ That is, we sought to evaluate the predictive performance of an NLP-derived model for SMM compared with the OB-CMI score. We hypothesized that compared with the current risk score, the NLP model would have similar sensitivity and positive predictive value for patients labeled as high risk for SMM.

## Methods

This was a retrospective diagnostic study of delivery encounters from a single academic medical center (Brigham and Women’s Hospital, Boston, Massachusetts) between July 1, 2016, and February 29, 2020. Delivery encounters were identified using the Z37 *International Statistical Classification of Diseases and Related Health Problems, Tenth Revision* diagnosis code. Delivery admissions between February and December 2018 were previously part of a prospective study that validated the use of the OB-CMI, a comorbidity-weighted risk score used during the delivery encounter to stratify a patient’s risk of SMM in clinical practice.^19^ In that validation study, nursing staff calculated the OB-CMI score based on the patient’s known comorbidities and pregnancy characteristics at admission; scores ranged from 0 to 15 (median of 1 [IQR, 0-3], with higher scores indicating increased risk of SMM) and were manually updated by a nurse at each shift change.^19^ Nursing staff were prompted to alert the attending physician for individuals with high scores (OB-CMI score >6 was used as the alert threshold in the validation study). This score was later retrospectively compared with cases of SMM, as defined by an individual panel review of each potential case of morbidity. The researchers demonstrated that the OB-CMI score predicted maternal morbidity well (area under the receiver operating characteristic curve [AUC], 0.83). The Mass General Brigham institutional review board approved both the original validation study and this retrospective analysis; informed consent of this study was waived because the study was retrospective, patient interaction was not involved, and the data were deidentified. This study followed the Transparent Reporting of a Multivariable Prediction Model for Individual Prognosis or Diagnosis (TRIPOD) reporting guideline.

We sought to evaluate the predictive performance of an NLP-derived model for SMM compared with the OB-CMI score. Because the OB-CMI score is first calculated at admission, we chose to analyze the free text in the clinician-written H&P note. We assumed that this note best reflected what the care team knew about the patient at the start of the delivery admission. We used a bag-of-words NLP model using monograms (single words). Each note was converted to a vector of the occurrence counts of individual words appearing within the body of a note. Standard preprocessing steps were used: removal of standardized text occurring in all notes, white-space normalization, removal of punctuation and letter cases, word stemming, and removal of stop words. Stop words are commonly occurring words within the English language (eg, *the*, *of*, and *at*); the SMART dictionary list in the stopwords package in R, version 4.0.2 (R Project for Statistical Computing) was used. To limit the sparseness of the data and improve analytic tractability, only terms that passed frequency thresholds (set a priori at ≥1% and ≤80%) were considered.

To generate the NLP model, we used delivery admission H&P notes filed in the institution’s EHR before (July 1, 2016, through January 31, 2018) and after (January 1, 2019, through February 29, 2020) the OB-CMI validation study^19^ (ie, the training set). For the rare encounters with multiple H&P notes (eg, individuals with transfers between services), the note with the earliest time stamp was used. For trainee-authored notes, the note often included an attending physician’s attestation. The analysis period ended before the start of the COVID-19 pandemic (February 29, 2020) to avoid any influence of COVID-19–related morbidity, which was not present during the initial OB-CMI validation project.

The primary outcome of interest was SMM (ie, unexpected outcomes of labor and delivery that are associated with significant short- or long-term consequences to a patient’s health), as defined by the Centers for Disease Control and Prevention.^26^ As a secondary outcome, we also examined the same outcome excluding transfusion (nontransfusion SMM), because transfusion is the most common component of the SMM composite outcome. *International Statistical Classification of Diseases and Related Health Problems, Tenth Revision* diagnosis and procedure codes from the delivery encounter were used to identify cases of morbidity. Of note, this outcome was similar, but not identical, to the outcome used in the original validation study,^19^ in which each case of potential morbidity was reviewed by an expert panel to assess whether SMM occurred by clinical consensus.

### Statistical Analysis

Individual least absolute shrinkage and selection operator (LASSO) models were used to assess the association between the words and their unscaled frequencies in the admission notes and the 2 outcomes, SMM and nontransfusion SMM (LASSO parameters: binomial distribution, α = 1; λ was selected to maximize the AUC via 10-fold cross-validation). A list of the highest-weighted terms for both outcomes is included in eTables 1 and 2 in the Supplement to enhance model interpretability. The NLP-based model was then tested on the H&P notes from the testing set (individuals included in the OB-CMI validation study). The AUCs were used to assess model discrimination and calibration plots for model calibration in the testing set; AUCs between the OB-CMI and NLP models were compared with the DeLong test.^27^

For translation to clinical practice, logistic regression models were used to assess the model performance using the following high-risk designations: having an OB-CMI score higher than 6 (in accordance with the original study^19^), having an NLP-based label, or meeting at least 1 of the 2 criteria (OB-CMI >6 or NLP-based label). The predicted probability threshold used to define the NLP-based label of high risk was selected in the training set such that it would yield the same screen-positive rate for an OB-CMI score higher than 6 (3.9%); this approach was used to facilitate direct comparability with the risk-stratification method currently in place (OB-CMI). Sensitivity-specificity plots were generated for both outcomes to display how these metrics compared across predicted probabilities and with the probability threshold corresponding to the NLP-based high-risk label. Agreement between an OB-CMI score higher than 6 and the NLP-based high-risk designation was assessed using the Cohen κ statistic. Model performance was assessed by comparing sensitivity and positive predictive value, which were the 2 metrics prioritized for this screening tool. Specificities and negative predictive values were also reported for comparison. To assess the value of the NLP-based model in addition to the OB-CMI, net reclassification indexes were calculated.^27^ The net reclassification index quantifies the value of additional information or factors to a predictive model, balancing the true- and false-positive and negative results.^28^

To characterize cases of model failure, 2 maternal-fetal medicine specialists (M.A.C., S.R.E.) manually reviewed the 10 false-positive and false-negative classifications with the highest predictive probabilities for SMM. These cases were reviewed to assess whether morbidity occurred and whether the admission notes contained information or tokens that imparted an increased risk for SMM. A detailed review of these cases is included in eTables 3 and 4 in the Supplement.

Baseline demographics—including age, self-reported race and ethnicity (Asian, Black, Hispanic, White, or other [multiple selected or undefined], collected to facilitate population comparisons with other hospitals or patient groups), and self-reported primary language—and the prevalence of SMM and nontransfusion SMM were compared between the training and testing sets using χ^2^ and 2-sided *t* tests, as appropriate. Because the OB-CMI score was known for only the testing set, we calculated and used Wilcoxon rank sum tests to compare a separate diagnosis code–based risk score (the Expanded Obstetric Comorbidity Score developed by Leonard et al^18^) between the training and testing sets. The score could be calculated for both SMM and nontransfusion SMM individually by summing the weights for different comorbidities and was used to understand the baseline prevalence and severity of comorbidities between the 2 groups.^18^

Two-sided *P* < .05 was considered statistically significant. Analyses were conducted using R, version 4.0.2, and Stata MP, version 16.1 (StataCorp LLC).^29,30^

## Results

This study included a total of 19 794 individuals who delivered at the study institution with an H&P note available for analysis. Of these, 4034 (20.4%) were included in the original prospective validation study of the OB-CMI; this group was excluded from the initial NLP model training and was used as the testing set, whereas the remaining 15 760 individuals (79.6%) composed the training set. Characteristics of participants in the training and testing sets are included in Table 1. There were no significant differences in age, race and ethnicity, or primary language between the 2 groups. In the training cohort, the mean (SD) age was 32.2 (5.2) years; 2143 (13.6%) identified as Asian, 1723 (10.9%) as Black, 1472 (9.3%) as Hispanic, 9164 (58.1%) as White, and 915 (5.8%) as other, with data missing for 343 (2.1%). A total of 14 275 individuals (90.6%) self-reported English as their primary language. In the testing cohort, the mean (SD) age of individuals was 32.3 (5.2) years; 548 (13.6%) identified as Asian, 435 (10.8%) as Black, 419 (10.4%) as Hispanic, 2304 (57.1%) as White, and 224 (5.6%) as other, with data missing for 104 (2.6%). A total of 3646 individuals (90.4%) self-reported English as their primary language. There was no significant difference in the median Expanded Obstetric Comorbidity Score between the training and testing sets for SMM (7 [IQR, 0-21] vs 7 [IQR, 0-22]; *P* = .16) or nontransfusion SMM (4 [IQR, 0-12] vs 4 [IQR, 0-13]; *P* = .20). The prevalence of maternal morbidity was also similar between the training and testing sets for SMM (468 [3.0%] vs 115 [2.9%]; *P* = .69) and nontransfusion SMM (208 [1.3%] vs 47 [1.2%]; *P* = .43).

**Table 1.** Characteristics of the Training and Testing Data Sets

Characteristic	Individuals (N = 19 794)^a^		*P* value
	Training (n = 15 760)	Testing (n = 4034)	
Age			
Mean (SD), y	32.2 (5.2)	32.3 (5.2)	.11
Missing	12 (0.1)	0	NA
Race and ethnicity			
Asian	2143 (13.6)	548 (13.6)	.15
Black	1723 (10.9)	435 (10.8)	
Hispanic	1472 (9.3)	419 (10.4)	
White	9164 (58.1)	2304 (57.1)	
Other^b^	915 (5.8)	224 (5.6)	
Missing	343 (2.1)	104 (2.6)	
Primary language			
English	14 275 (90.6)	3646 (90.4)	.61
Not English	1473 (9.4)	388 (9.6)	
Missing	12 (0.1)	0	
Expanded Obstetric Comorbidity Index, median (IQR)^c^			
SMM	7 (0-21)	7 (0-22)	.16
Nontransfusion SMM	4 (0-12)	4 (0-13)	.20
Severe maternal morbidity			
Including transfusion	468 (3.0)	115 (2.9)	.69
Excluding transfusion	208 (1.3)	47 (1.2)	.43

Abbreviations: NA, not applicable; SMM, severe maternal morbidity.

^a^ Data are expressed as the number (percentage) of individuals unless otherwise indicated.

^b^ Other included multiple or undefined races and ethnicities.

^c^ Derived by Leonard et al.^18^

An OB-CMI score higher than 6 was used clinically in the original validation study^19^ to designate individuals as having a high risk of morbidity. At admission, 159 of 4034 individuals (3.9%) in the training set had a score higher than 6. In this group, the prevalence of maternal morbidity was notably higher than that in the group with scores of 6 or lower: 28 of 159 (17.6%) vs 87 of 3875 (2.2%) for SMM (*P* < .001) and 17 of 159 (10.7%) vs 30 of 3875 (0.8%) for nontransfusion SMM (*P* < .001).

**Figure 1.**
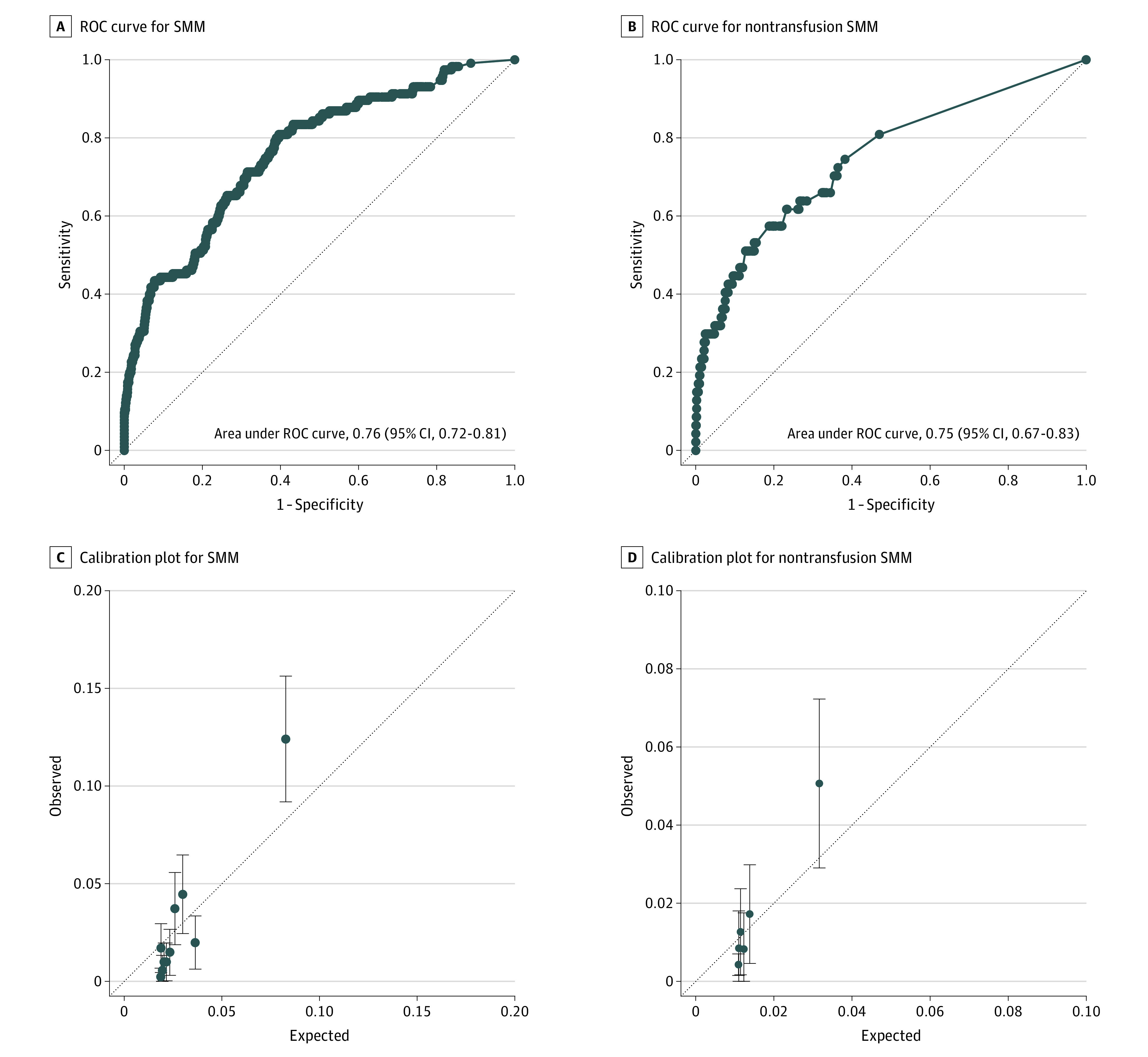
Receiver Operating Characteristic (ROC) Curves and Calibration Plots of the Natural Language Processing Model for Severe Maternal Morbidity (SMM) and Nontransfusion SMM A and B, Diagonal dotted lines indicate an area under the ROC curve of 0.5. C and D, Circles indicate point estimates; whiskers, 95% CIs; and diagonal dotted lines, perfect calibration.

**Figure 2.**
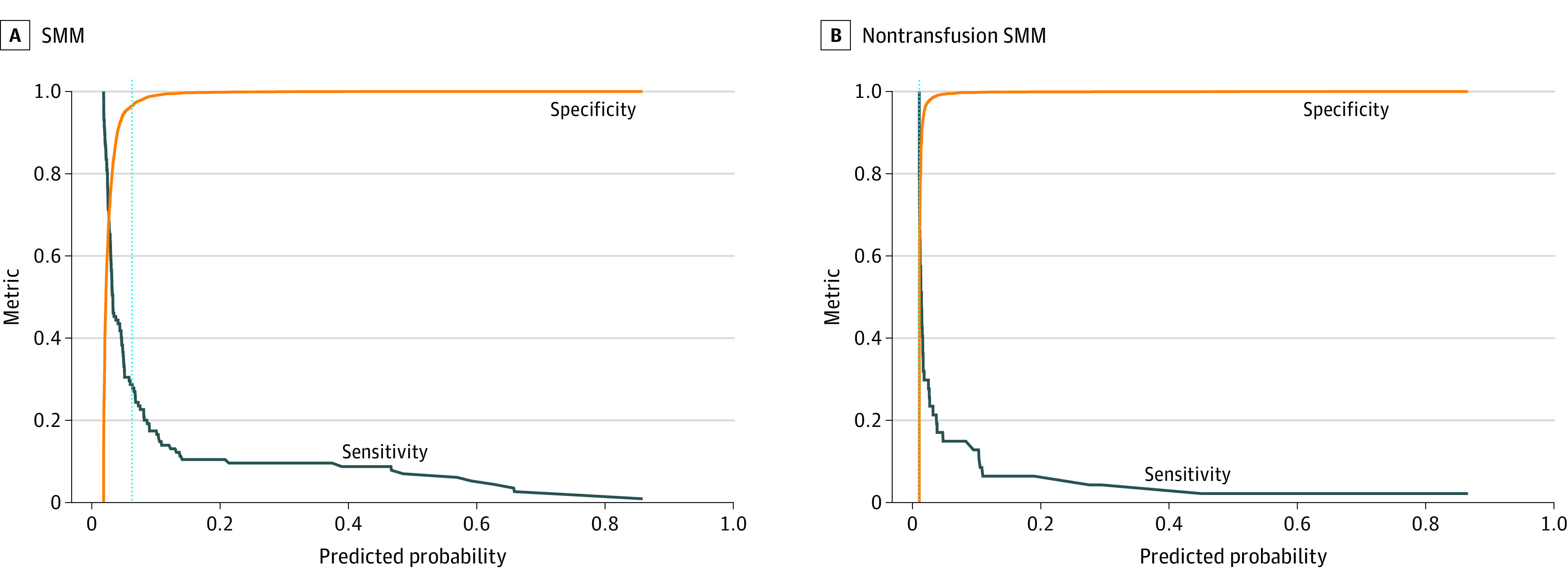
Sensitivity-Specificity Plots Across the Range of Natural Language Processing Predicted Probabilities and the Probability Threshold Used to Identify Individuals at High Risk of Severe Maternal Morbidity (SMM) Vertical dotted line indicates the screen-positive threshold, the high-risk designation.

The NLP model was built from an original vocabulary of 78 695 unique words that occurred within the 15 760 admission notes in the training set; the final pruned vocabulary contained 2783 tokens after preprocessing. The occurrence counts of each word were used to generate a collective probability for maternal morbidity for each note. The performance characteristics of the NLP-based model developed in the training set and applied to the testing set are shown in Figure 1. The AUCs (Figure 1A and B) for the prediction of SMM and nontransfusion SMM in the testing set were 0.76 (95% CI, 0.72-0.81) and 0.75 (95% CI, 0.67-0.83), respectively. These were comparable with the AUCs for OB-CMI to predict SMM (0.74; 95% CI, 0.70-0.79; *P* = .53) and nontransfusion SMM (0.78; 95% CI, 0.72-0.86; *P* = .46). The calibration plots (Figure 1C and D) revealed that the models were not well calibrated across all predicted probabilities, but they did distinguish a subset of individuals at significantly higher risk. Figure 2 shows the comparison between sensitivity and specificity across all predicted probabilities from the NLP model and the screen-positive threshold used to designate individuals at highest risk.

**Figure 3.**
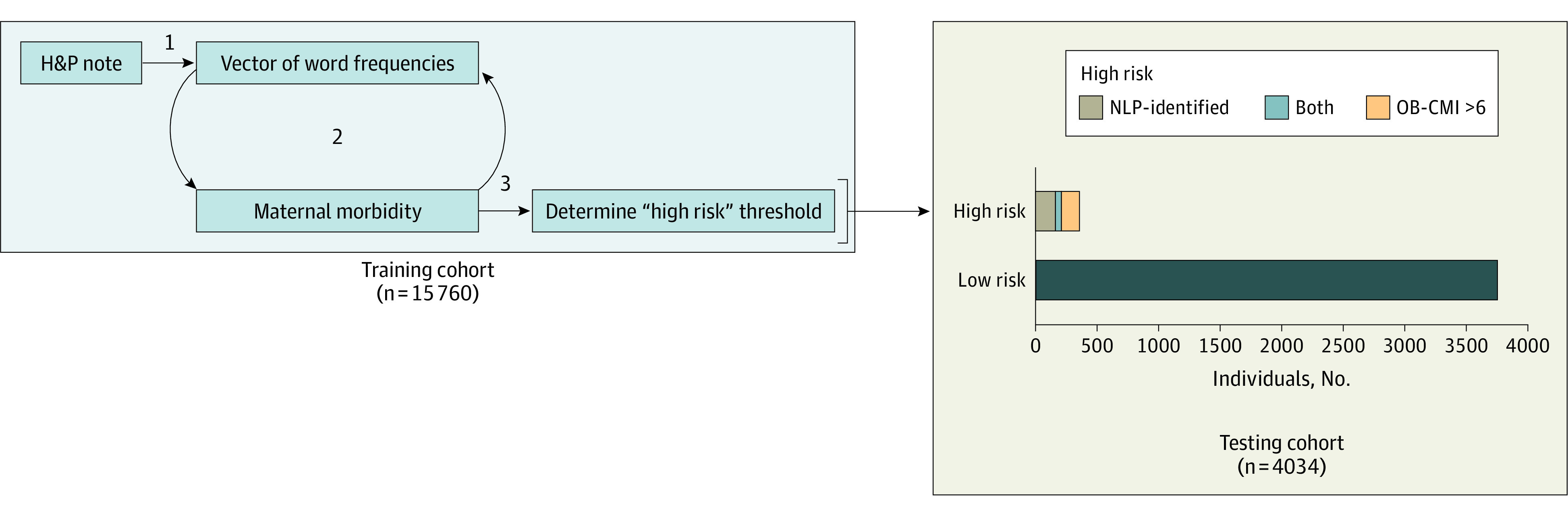
Visual Summary of the Natural Language Processing (NLP) Model Development in the Training and Testing Cohorts Step 1 involved converting history and physical (H&P) notes into a vector of word frequencies in a training cohort and then using least absolute shrinkage and selection operator models to assess each note’s association with severe maternal morbidity (step 2). In step 3, a screen-positive threshold was applied to designate individuals as high risk. This threshold was evaluated in a testing cohort and compared with the high-risk designation using the Obstetric Comorbidity Index (OB-CMI). In the testing cohort, 170 individuals were classified as high risk by the NLP-based method and 159 using the OB-CMI threshold greater than 6; 42 were classified as high risk by both methods.

Figure 3 summarizes how the OB-CMI and NLP-based risk-stratification methods compared. Of the 4034 individuals in the testing set, 170 (4.2%) were labeled as high risk by the NLP model. Forty-two of 4034 individuals (1.0%) were labeled as high risk for SMM using both the NLP and the OB-CMI screen-positive thresholds (κ = 0.22), whereas 287 of 4034 (7.1%) were labeled high risk using either definition. Of note, the prevalence of SMM among the 42 individuals labeled as high risk by both approaches was 42.9% (18 individuals). Similar overlaps were seen for nontransfusion SMM: 33 of 4034 individuals (0.8%) were labeled high risk for nontransfusion SMM using both approaches, and 318 of 4034 (7.9%) were labeled high risk using either approach. The prevalence of nontransfusion SMM among the 33 individuals labeled as high risk by both approaches was 30.3% (10 individuals).

Table 2 shows the test characteristics for each high-risk designation approach for SMM: OB-CMI score higher than 6, NLP-based threshold, and the inclusive combination (OB-CMI and NLP) of both approaches (ie, either OB-CMI score >6 or highest-predicted NLP risk). The inclusive OB-CMI and NLP approach had the highest sensitivity: 37.4% (combined) vs 24.4% (OB-CMI alone) vs 28.7% (NLP alone). The positive predictive value was slightly lower in the combined approach: 15.0% (combined) vs 19.4% (NLP) vs 17.6% (OB-CMI). The net reclassification index for the addition of the NLP to the OB-CMI screen-positive approach was positive (0.104), meaning that there was a net benefit. For SMM, the NLP addition correctly classified an additional 15 individuals as high risk among 115 individuals who had SMM (13.0%), whereas it only newly misclassified as high risk 103 of the 3919 individuals who did not have SMM (2.6%). Confusion matrixes for the NLP, OB-CMI, and combined OB-CMI and NLP risk designations for SMM are shown in eTables 5 to 7 in the Supplement.

**Table 2.** Comparison of Model Performance Characteristics for the OB-CMI and NLP Models and the Combination Approach in Identifying Individuals at High Risk for SMM or Nontransfusion SMM

Model characteristic	Metric, %					
	SMM			Nontransfusion SMM		
	OB-CMI	NLP	OB-CMI and NLP	OB-CMI	NLP	OB-CMI and NLP
Screen-positive rate	3.9	4.2	7.1	3.9	4.1	7.2
Sensitivity	24.4	28.7	37.4	36.2	29.8	44.7
Positive predictive value	17.6	19.4	15.0	10.7	8.4	6.8
Specificity	96.7	96.5	93.8	96.4	96.2	93.2
Negative predictive value	97.8	97.9	98.1	99.2	99.2	99.3

Abbreviations: NLP, natural language processing; OB-CMI, Obstetric Comorbidity Index; SMM, severe maternal morbidity.

Similar findings were demonstrated for nontransfusion SMM, as shown in Table 2. The combined approach also had the highest sensitivity: 44.7% (combined) vs 36.2% and 29.8% for the OB-CMI and NLP approaches, respectively. The positive predictive value was slightly lower in the combined approach: 6.8% (combined) vs 10.7% and 8.4% in the OB-CMI and NLP approaches, respectively. For nontransfusion SMM, the net reclassification index for the addition of the NLP to the OB-CMI screen-positive approach was also positive (0.052). The NLP addition correctly classified an additional 4 individuals as high risk among 47 individuals who had nontransfusion SMM (8.5%), whereas it only newly misclassified 129 individuals as high risk among the 3987 individuals who did not have nontransfusion SMM (3.2%). Confusion matrixes for the NLP, OB-CMI, and combined OB-CMI and NLP risk designations for nontransfusion SMM are shown in eTables 8 to 10 in the Supplement.

The failure analysis (eTables 3 and 4 in the Supplement) revealed that 10 of 10 false-positive cases (100%) contained tokens and content that conveyed an increased risk for SMM: 8 of 10 (80%) were associated with risk for or history of placenta previa and/or accreta. None of these cases had diagnosis or procedure codes that met criteria for SMM. In a review of the false-negative cases, all patients had conditions that are known to increase the risk of delivery-related complications (eg, multiple gestations, hypertension, and trial of labor after cesarean delivery); the predicted probability of SMM ranged from 4.8% to 6.0% (compared with the baseline incidence of 2.9%).

## Discussion

The results of this study demonstrated that automated text processing of admission H&P notes performed as well as the study institution’s current standard practice for SMM risk stratification, which is a validated score manually calculated by clinical staff at the start of the admission. The NLP approach had similar performance for the outcomes of maternal morbidity that included and excluded transfusion, which is the most common component in the Centers for Disease Control and Prevention–defined SMM metric.^26^ Although the overall NLP model calibration was poor across the range of all predicted probabilities, the approach identified a subset of individuals at significantly higher risk of morbidity; this finding likely reflects that most pregnant individuals were healthy and had no identifiable factors associated with morbidity. This study demonstrated that the NLP approach identified a somewhat different subset of individuals as being at high risk for morbidity, including an additional 15 of the 115 individuals who had SMM. We hypothesize that this result may have been associated with (1) the OB-CMI score threshold of higher than 6 being used to define high risk in the original validation study (eg, preeclampsia with severe features [OB-CMI score weight of 5] would not have been considered high risk unless other risk factors were present) and (2) the relative inflexibility of a tool that has limited inputs by design to facilitate implementation.^19^ Among individuals who met both the NLP and the OB-CMI criteria, 42.9% experienced SMM, highlighting a small group at exceptionally high risk of an adverse outcome.

Most labor and delivery units rely on risk-stratification systems that require manually input data.^19^ Such tools require clinical staff to perform additional non–patient-facing tasks at the bedside and collate information from multiple places within the EHR; this task burden is balanced with the potential benefits of having an informed team that is prepared to mitigate and manage potential adverse events.^31,32^ The application of machine learning and artificial intelligence, such as NLP, presents an innovative opportunity to improve clinical care, such as maternal risk-stratification methods, without generating additional work for health care professionals. Although these NLP-based analytic approaches are not routinely used within EHR systems yet, they are commonly used in nonmedical applications (eg, internet searches) and have potential translatability to health care.^21,22,33,34^ Because clinical documentation is generated predictably with each hospital admission, successful NLP-based analyses may ultimately have widespread generalizability across EHR platforms.

We demonstrated the potential application of NLP in routine clinical documentation for risk stratification of maternal morbidity. The next steps of this work involve the use of this NLP-augmented risk-stratification approach in a pilot study to understand its performance in practice and how this additional information may change behavior of health care professionals and teams as it relates to risk preparedness. To mirror the workflow and implementation of the OB-CMI tool during the original prospective study,^19^ we chose a screen-positive rate for the NLP model that was identical to the rate when using the OB-CMI tool. The screen-positive threshold (for both the OB-CMI and the NLP models) could be tailored for individual birthing units to align model performance with the resources and personnel available in a given unit.

### Strengths and Limitations

This study has strengths, including its large sample size, prospective manual assignment of the OB-CMI score, and the relative simplicity of the NLP methods, all of which may facilitate future implementation into practice. The study used clinical documentation that best reflected what was known by the clinical team at the start of the delivery encounter, which contrasts with diagnosis code–derived scores. This admission-based approach is also relevant for determining risk-appropriate care.

This study also has limitations. The main limitation is its population sample, which was derived from patients delivering at a single large academic center. Although health care professionals at the center include obstetricians, certified nurse midwives, and trainees, all of whom may author admission notes, the documentation practices may reflect institutional practices and local vernacular. Prior NLP work has demonstrated that the individual vocabularies generated from a single institution may limit the generalizability to other applications or centers; future external validation studies should be conducted.^35^ In some instances, H&P notes may have been written after an outcome had already occurred; to limit the likelihood of this, we restricted the analysis to the earliest written note before delivery during the encounter. This approach used monograms (single words), which may fail to recognize concepts in more complex sentence structures (eg, negation); whereas prior work by some of us showed that bigram and trigram models did not improve model performance,^20^ more advanced NLP methods could be used in future studies. As with all models, performance characteristics are dependent on the probability thresholds used to define risk categories; the sensitivity-specificity plots were included to show how these metrics would change across all predictive probabilities for the NLP model. Risk of morbidity can evolve during admission as additional data become available; the addition of intrapartum factors in future studies may allow for a more tailored risk estimate that is updated throughout a patient’s delivery admission. In addition, the outcome (SMM) was defined using diagnosis and procedure codes, which inherently lack temporality, as is true of all population-based studies using encounter-level data; it is likely some individuals had conditions classified as SMM at admission.

## Conclusions

In this diagnostic study, the NLP-based morbidity risk-stratification tool using standard clinical admission documentation performed as well as the validated OB-CMI tool, which is currently the institutional standard. The OB-CMI and NLP tools used together had higher sensitivity and a similar positive predictive value compared with either tool used alone. The net outcome of adding the NLP-based model to the current approach was that more individuals who ultimately had morbidity were correctly classified as high risk. These findings add to a growing body of work demonstrating the potential applications of advanced analytics using EHR data to improve patient care. Future prospective research is needed to validate this type of approach in clinical practice and to study key aspects that may influence wider adoption of NLP, including the ability to explain the models to patients and physicians and maintain (eg, avoid concept drift) and implement them.

## Notes

Supplement. **eTable 1.** Table of Highest Weighted Terms for the Prediction of Severe Maternal Morbidity **eTable 2.** Table of Highest Weighted Terms for the Prediction of Nontransfusion Severe Maternal Morbidity **eTable 3.** Analysis of the False-Positive Predictions for Severe Maternal Morbidity **eTable 4.** Analysis of the False-Negative Predictions for Severe Maternal Morbidity **eTable 5.** Confusion Matrix for the NLP-based Method for Classifying Risk for Severe Maternal Morbidity **eTable 6.** Confusion Matrix for the OB-CMI-based Method for Classifying Risk for Severe Maternal Morbidity **eTable 7.** Confusion Matrix for the Combined NLP and OB-CMI–based Method for Classifying Risk for Severe Maternal Morbidity **eTable 8.** Confusion Matrix for the NLP-based Method for Classifying Risk for Nontransfusion Severe Maternal Morbidity **eTable 9.** Confusion Matrix for the OB-CMI-based Method for Classifying Risk for Nontransfusion Severe Maternal Morbidity **eTable 10.** Confusion Matrix for the Combined NLP and OB-CMI–based Method for Classifying Risk for Nontransfusion Severe Maternal Morbidity
