# Supplementary material for: Comparison of Natural Language Processing of Clinical Notes With a Validated Risk-Stratification Tool to Predict Severe Maternal Morbidity
Source: JAMA Netw Open. 2022 Oct 5;5(10):e2234924. doi: 10.1001/jamanetworkopen.2022.34924 (PMC9535539; doi:10.1001/jamanetworkopen.2022.34924)
Supplement: Supplement. — eTable 1. Table of Highest Weighted Terms for the Prediction of Severe Maternal Morbidity eTable 2. Table of Highest Weighted Terms for the Prediction of Nontransfusion Severe Maternal Morbidity eTable 3. Analysis of the False-Positive Predictions for Severe Maternal Morbidity eTable 4. Analysis of the False-Negative Predictions for Severe Maternal Morbidity eTable 5. Confusion Matrix for the NLP-based Method for Classifying Risk for Severe Maternal Morbidity eTable 6. Confusion Matrix for the OB-CMI-based Method for Classifying Risk for Severe Maternal Morbidity eTable 7. Confusion Matrix for the Combined NLP and OB-CMI–based Method for Classifying Risk for Severe Maternal Morbidity eTable 8. Confusion Matrix for the NLP-based Method for Classifying Risk for Nontransfusion Severe Maternal Morbidity eTable 9. Confusion Matrix for the OB-CMI-based Method for Classifying Risk for Nontransfusion Severe Maternal Morbidity eTable 10. Confusion Matrix for the Combined NLP and OB-CMI–based Method for Classifying Risk for Nontransfusion Severe Maternal Morbidity [file jamanetwopen-e2234924-s001.pdf]

Supplementary Online Content

Clapp MA, Kim E, James KE, et al. Comparison of natural language processing of clinical notes with a validated risk-stratification tool to predict severe maternal morbidity. *JAMA Netw Open*. 2022;5(10):e2234924. doi:10.1001/jamanetworkopen.2022.34924

eTable 1. Table of Highest Weighted Terms for the Prediction of Severe Maternal Morbidity
eTable 2. Table of Highest Weighted Terms for the Prediction of Nontransfusion Severe Maternal Morbidity
eTable 3. Analysis of the False-Positive Predictions for Severe Maternal Morbidity
eTable 4. Analysis of the False-Negative Predictions for Severe Maternal Morbidity
eTable 5. Confusion Matrix for the NLP-based Method for Classifying Risk for Severe Maternal Morbidity
eTable 6. Confusion Matrix for the OB-CMI-based Method for Classifying Risk for Severe Maternal Morbidity
eTable 7. Confusion Matrix for the Combined NLP and OB-CMI–based Method for Classifying Risk for Severe Maternal Morbidity
eTable 8. Confusion Matrix for the NLP-based Method for Classifying Risk for Nontransfusion Severe Maternal Morbidity
eTable 9. Confusion Matrix for the OB-CMI-based Method for Classifying Risk for Nontransfusion Severe Maternal Morbidity
eTable 10. Confusion Matrix for the Combined NLP and OB-CMI–based Method for Classifying Risk for Nontransfusion Severe Maternal Morbidity

This supplementary material has been provided by the authors to give readers additional information about their work.

© 2022 Clapp MA et al. *JAMA Network Open*.

eTable 1: Table of Highest Weighted Terms for the Prediction of Severe Maternal Morbidity

Stemmed Term	Coefficient
cv	0.122
point	0.125
mag	0.128
sgpt	0.129
site	0.134
complex	0.142
safet	0.151
fellow	0.158
llp	0.165
id	0.171
presum	0.171
hematolog	0.205
transfus	0.207
co2	0.213
chewabl	0.214
stat	0.233
code	0.255
cathet	0.281
coag	0.461
hysterectom	0.571

© 2022 Clapp MA et al. *JAMA Network Open*.

eTable 2: Table of Highest Weighted Terms for the Prediction of Nontransfusion Severe Maternal Morbidity

Stemmed Term	Coefficient
latex	0.053
bps	0.073
id	0.099
concern	0.102
mitral	0.102
lupus	0.104
transfer	0.145
sickl	0.151
cosign	0.153
accreta	0.183
addit	0.185
fellow	0.199
coag	0.221
cv	0.240
site	0.276
lfts	0.330
co2	0.406
sgpt	0.414
hysterectom	0.491
code	0.611

© 2022 Clapp MA et al. *JAMA Network Open*.

eTable 3: Analysis of the False-Positive Predictions for Severe Maternal Morbidity

Case Number	High-risk Label	SMM	OB-CMI Score	Case Vignette
1	1	0	6	Delivered preterm in the setting of a bleeding previa with concern for possible accreta; s/p cesarean delivery (no accreta)
2	1	0	6	Delivered preterm for pre-eclampsia with severe features; history of renal artery aneurysm s/p repair; s/p cesarean delivery
3	1	0	9	Delivered at term with diabetes, hypertension; history of pre-eclampsia and stroke
4	1	0	4	Delivered at term; history of postpartum hemorrhage and focal accreta requiring blood transfusion
5	1	0	1	Delivered preterm for abruption; fetus with cardiac anomaly
6	1	0	6	Delivered at term via scheduled cesarean delivery; history of myomectomy
7	1	0	0	Delivered at term; history of retained placenta/focal accreta required dilation and curettage
8	1	0	1	Delivered at term; history of placenta accreta and postpartum hemorrhage requiring blood transfusion
9	1	0	6	Delivered preterm in the setting of bleeding with placenta previa
10	1	0	4	Delivered preterm in the setting of premature rupture of membranes; history of accreta / previa in prior pregnancy

© 2022 Clapp MA et al. *JAMA Network Open*.

eTable 4: Analysis of the False-Negative Predictions for Severe Maternal Morbidity

Case Number	High-risk Label	SMM	OB-CMI Score	Case Vignette
1	0	1	2	Multiple gestation delivered preterm; delivery complicated by postpartum hemorrhage requiring transfusion
2	0	1	5	Vaginal birth after cesarean delivery at term gestation; delivery complicated by postpartum hemorrhage requiring transfusion
3	0	1	3	Scheduled cesarean delivery at term, experienced syncope
4	0	1	5	Multiple gestation who presented with hypertension and shortness of breath; delivery admission complicated by pulmonary edema
5	0	1	9	Multiple gestation who presented with pre-eclampsia with severe features; delivery complicated by postpartum hemorrhage requiring transfusion and pulmonary edema
6	0	1	4	Multiple gestation delivered at term; delivery complicated by postpartum hemorrhage requiring transfusion
7	0	1	1	Multiple gestation with a history of an intracranial aneurysm who underwent cesarean delivery
8	0	1	6	Vaginal delivery at term after induction for hypertension; postpartum course complicated by acute kidney injury
9	0	1	16	Preterm delivery for shortness of breath in a patient with a history of cardiomyopathy and worsening chronic hypertension
10	0	1	6	Preterm delivery after presenting with pre-eclampsia with severe features; postpartum course complicated by pulmonary edema and acute kidney injury

© 2022 Clapp MA et al. *JAMA Network Open*.

eTable 5: Confusion Matrix for the NLP-based Method for Classifying Risk for Severe Maternal Morbidity

	No Severe Maternal Morbidity	Severe Maternal Morbidity
Not High Risk	3,782	82
High Risk	137	33

© 2022 Clapp MA et al. *JAMA Network Open*.

eTable 6: Confusion Matrix for the OB-CMI-based Method for Classifying Risk for Severe Maternal Morbidity

	No Severe Maternal Morbidity	Severe Maternal Morbidity
Not High Risk	3,788	87
High Risk	131	28

© 2022 Clapp MA et al. *JAMA Network Open*.

eTable 7: Confusion Matrix for the Combined NLP and OB-CMI–based Method for Classifying Risk for Severe Maternal Morbidity

	No Severe Maternal Morbidity	Severe Maternal Morbidity
Not High Risk	3,675	72
High Risk	244	43

© 2022 Clapp MA et al. *JAMA Network Open*.

eTable 8: Confusion Matrix for the NLP-based Method for Classifying Risk for Nontransfusion Severe Maternal Morbidity

	No Severe Maternal Morbidity	Severe Maternal Morbidity
Not High Risk	3,835	33
High Risk	152	14

© 2022 Clapp MA et al. *JAMA Network Open*.

eTable 9: Confusion Matrix for the OB-CMI-based Method for Classifying Risk for Nontransfusion Severe Maternal Morbidity

	No Severe Maternal Morbidity	Severe Maternal Morbidity
Not High Risk	3,845	30
High Risk	142	17

© 2022 Clapp MA et al. *JAMA Network Open*.

eTable 10: Confusion Matrix for the Combined NLP and OB-CMI–based Method for Classifying Risk for Nontransfusion Severe Maternal Morbidity

	No Severe Maternal Morbidity	Severe Maternal Morbidity
Not High Risk	3,716	26
High Risk	271	21

© 2022 Clapp MA et al. *JAMA Network Open*.
